# Recognizing Emily and Latisha: Inconsistent Effects of Name Stereotypicality on the Other-Race Effect

**DOI:** 10.3389/fpsyg.2018.00486

**Published:** 2018-04-13

**Authors:** Marleen Stelter, Juliane Degner

**Affiliations:** Department of Social Psychology, Universität Hamburg, Hamburg, Germany

**Keywords:** other-race effect, first names, face perception, person perception, stereotypes

## Abstract

A person’s name may activate social category information, which has been shown to lead to stereotyping and discrimination in various contexts. However, no previous research has investigated the influence of names on more basic processes of person perception. We present a set of seven experimental studies examining the influence of names on face recognition, namely, on the other-race effect (i.e., the relative difficulty to recognize outgroup faces). White-American participants completed online recognition tasks with White ingroup faces and Black or Chinese outgroup faces. Outgroup faces were presented with typical outgroup names versus typical White names; White faces were presented with typical White names versus infrequent names. We expected better recognition of outgroup faces with typical White names compared to outgroup faces with typical outgroup names. Employing an internal meta-analysis, we observe overall evidence of a small but significant effect (*d*_*z*_ = 0.11). However, the pattern of results across the seven studies is inconsistent. Given that particularly the high-powered pre-registered studies did not show an effect, we suggest that the effect should be interpreted with caution. We discuss that a small effect may still have important implications for real life as well as for theories of the ORE, emphasizing the importance of future research regarding the influence of name typicality on inter-group face perception.

## Introduction

Imagine that you are about to meet someone for the first time, and the only information you have in advance is that the person’s given name is Dylan. Whom do you expect to meet? Would you be surprised to find out that Dylan was African American? Numerous social psychological studies document that merely knowing a person’s name activates a wealth of expectations, for example, regarding the individual’s ethnic group membership or socio-economic status (e.g., Young et al., 1993; Fryer and Levitt, 2004). The present research investigates if and to what extent perceived *ethnic typicality* of given names influences memory for faces. Specifically, we examine if typical versus untypical names modulate the so-called other-race effect (ORE)—the well-documented memory effect that people are better at recognizing ingroup faces compared to outgroup faces.

We investigate this question in the context of the United States, where many given names are distinctively associated with different ethnic groups (see Tzioumis, 2015). For example, while common English names such as ‘Emily’ or ‘Brad’ are perceived to be typical White (Cotton et al., 2014), other names such as ‘Tyrone’ and ‘Latisha’ are almost exclusively chosen by African American parents (Fryer and Levitt, 2004). Previous research has shown that names that are associated with minority groups are evaluated more negatively (e.g., Greenwald et al., 1998) and activate group-specific stereotypes (Holbrook et al., 2016). Furthermore, numerous studies document name-based discrimination in many different domains (e.g., Greenwald et al., 1998; Bertrand and Mullainathan, 2004; Carpusor and Loges, 2006; Ahmed and Hammarstedt, 2008; Cotton et al., 2008; Pager and Shepherd, 2008; Okonofua and Eberhardt, 2015; Hanson et al., 2016; Holbrook et al., 2016). However, to our knowledge, no research has investigated the influence of names on more basic processes of person perception. We suggest that names may also influence cognitive processes of person perception, such as memory for faces. In particular, we suggest that name typicality may influence the magnitude of the ORE.

The ORE is a very robust effect that has been replicated many times across many different cultural contexts and intergroup settings (see Meissner and Brigham, 2001, for a meta-analysis)—as have its drastic consequences, for example, in the legal context (Wilson et al., 2013). The ORE is assumed to be caused by an interplay of visual expertise and social-cognitive factors (see Young et al., 2012 for a comprehensive review of theories). While theories of visual expertise emphasize the role of (reduced) contact with outgroup members, and thus, a lack of expertise for explaining impaired face recognition (e.g., Brigham and Malpass, 1985; Hancock and Rhodes, 2008), social-cognitive theories emphasize the additional role of motivational factors and social categorization processes (e.g., Levin, 2000; Hugenberg et al., 2013). Particularly, social-cognitive theories assume that memory for outgroup faces is impaired because outgroup faces are processed on a *categorical* level (focusing on shared category information, such as skin color), whereas ingroup faces are processed on an *individual* level (focusing on what makes the face unique, e.g., Fiske and Neuberg, 1990; Cloutier and Macrae, 2007). These differences in categorical versus individuated processing are assumed to (partly) depend on the perceiver’s motivation to individuate a face during encoding (Hugenberg et al., 2013). Supporting this idea, previous research has shown that the ORE is reduced or even disappears in certain social contexts (see Kawakami et al., 2017, for an extensive review). For example, the ORE has been found to be reduced for high-status outgroup members (Shriver et al., 2008), in situations of outcome dependency (Baldwin et al., 2012), if there is a shared group identity (Van Bavel and Cunningham, 2012), or if participants are instructed to individuate outgroup faces (Hugenberg et al., 2007; but see Pica et al., 2015).

The present research expands on the idea that the ORE can be modulated by social context information: We explore if ethnic typicality of given names may influence memory for outgroup faces. Specifically, we investigate if White Americans participants are better at recognizing Black and Asian faces if they have typical White names compared to if they have typical Black or Asian names. We suggest that there may be several different mechanisms of how name typicality may influence the ORE.

One potential mechanism may be that White names are perceived as untypical for Black or Asian individuals and may thus violate participants’ expectations, because they are *inconsistent* with the initial face categorization. This perceived inconsistency may affect memory in two different ways: First, it is possible that, for example, Black faces with White names may become more salient and may thus benefit from a general inconsistency-memory advantage (see Dijksterhuis and Van Knippenberg, 1995; but see Sherman et al., 1998). Second, based on social cognitive models of person perception (e.g., Fiske and Neuberg, 1990), the perceived inconsistency may also initiate a more elaborate and individuated face-processing, which may in turn enhance memory (see Hugenberg et al., 2013). Conversely, we suggest that Black or Asian faces carrying typical Black or Asian names may be consistent with White American participants’ expectations and may thus lead to category-based face processing, causing a classic ORE.

Another potential mechanism may be that White names versus Black names may lead to different top-down processing of outgroup faces (see also Kawakami et al., 2017). For example, it is possible that Black individuals with White names may be perceived as having a higher *social status* than Black individuals with Black names—given that previous research has shown that Black names are associated with stereotypes of lower status (Fryer and Levitt, 2004; Cotton et al., 2008). Sub-categorizing Black individuals with White names as members of a higher-status group may also enhance participants’ motivation to individuate their faces (see Shriver et al., 2008). Similarly, it is also feasible that Black names may serve as an additional racial marker. As a consequence, faces of Black individuals with White names may be perceived as *less prototypical* than the faces of Black individuals with Black names. Thus, the effect of Black versus White names on perception of Black faces may be similar to effects of other racial markers on racially ambiguous faces (e.g., Maclin and Malpass, 2001; Eberhardt et al., 2003; Pauker et al., 2009; Hourihan et al., 2013). In short, drawing on previous findings from the memory literature as well as on social-cognitive theories of the ORE, it is conceivable that name typicality may modulate memory for outgroup faces. Finding an effect of name typicality on the magnitude of the ORE may add to the ORE literature in an important way, by providing further evidence for the social malleability of the ORE, thus, corroborating the assumptions of social-cognitive theories of the ORE.

We investigated the influence of name typicality on the ORE in a series of seven studies, examining if participants are better at remembering outgroup faces with typical ingroup names compared to outgroup faces with typical outgroup names. All studies included only White American participants, thus, Black or Chinese faces were outgroup faces and White faces were always ingroup faces. All studies employed an old/new recognition task, in which participants memorized a series of ingroup and outgroup faces.

During the study phase, outgroup faces were presented either with typical outgroup names or typical White names; White faces were presented either with typical White names or with infrequent names that were neither typical for Black, Chinese, nor White Americans. We included infrequent names as a control condition, with the following rationale: Because of different population sizes (i.e., African Americans constitute ∼13% of the United States population and Chinese Americans constitute ∼1.2% of the United States population), typical Black names and Chinese names are overall less frequent than typical White names and may thus be generally less familiar, especially for White participants. We therefore selected a set of infrequent names matching the lower frequencies of typical Black names and Chinese names (see also Cotton et al., 2014), in order to account for potential effects of name familiarity (i.e., impaired memory due to reduced familiarity with infrequent Black names). After the study phase, participants were asked to recognize either the faces or the names. As our main research question addresses the influence of given names on face recognition, the following analyses and discussion focus only on results of the face recognition conditions. Results of the name recognition conditions are reported in the Supplementary Material.

All seven studies were conducted online with participants recruited via Amazon Mechanical Turk (MTurk) or Prolific (only Study 6). First, we conducted a pilot study with male Black and White faces, verifying that the procedure of the old/new recognition task was applicable to an online setting. Study 1 was a direct replication of the pilot study; Study 2 aimed at conceptually replicating effects with female Black and White faces; Study 3 aimed at conceptually replicating effects with male Chinese and White faces; Studies 4 and 5 were direct replications of Studies 1 and 2 with larger sample sizes, in order to obtain sufficient test power to test for small effects. Study 6 was another high-powered direct replication of the effect of Studies 1 and 4. Studies 1, 4, 5, and 6 were pre-registered via the Open Science Framework (OSF). Preregistrations, experimental procedures, raw data files, and analysis scripts are publically accessible online on OSF^1^. We report how we determined our sample sizes, all data exclusions, all manipulations, and all measures of the seven studies.

We pre-registered four hypotheses: The first two hypotheses related to face recognition performance; two further hypotheses are related to name recognition performance and are reported in the Supplementary Tables S3, S4). As a first hypothesis, we expected a classic ORE, with better recognition of White faces compared to Black faces. As a second hypothesis, we expected the ORE in face recognition to be modulated by name typicality. Specifically, we hypothesized that White American participants recognize Black or Chinese faces presented with typical White names better than Black or Chinese faces with typical Black or Chinese names. We analyzed these hypotheses for the individual studies via paired *t*-test and generalized linear mixed-effects models (see Supplementary Material), as well as via internal meta-analyses, summarizing effects across all studies. These so-called *mini-metas* have been proposed to be advantageous, because they allow computation of more precise effect size estimates (see Cumming, 2014; Goh et al., 2016; Lakens and Etz, 2017).

## Pilot Study and Studies 1 and 2

### Method

#### Participants

Participants were recruited via MTurk with the selection criterion that their location was in the United States. The Pilot study had the additional selection criteria that HIT approval rates were at least 95% percent and that the number of approved HITs was greater than 5000; for Studies 1 and 2, HIT approval rates had to be at least 98% percent. Participants were paid $1.25 US Dollar ($0.50 US Dollar for the Pilot study). Participants who failed an instructional manipulation check on the second page of the experiment were precluded from taking the experiment. In line with the pre-registrations, we subsequently excluded participants who did not self-categorize as White or who did not perform above chance in the recognition test (proportion of correct responses <0.50). Targeted sample sizes of *N* = 61 for the face recognition condition in Studies 1 and 2 were obtained via an *a priori* power analysis for a one-tailed paired *t*-test based on an effect size of *d*_*z*_ = 0.38 for hypothesis 2 with a test power of 1-β = 0.85 and α = 0.05. This effect size originated from the pilot study, but unfortunately, had been calculated inaccurately, which we noticed only after data collection for Studies 1, 2, and 3 were completed (see also preregistration of Study 1 on OSF). We over-powered both studies, given that it was very difficult to recruit the exact number of participants meeting the inclusion criteria. Final sample sizes, number of excluded participants, and demographic information are reported in **Table 1** and Supplementary Table S2.

**Table 1:** Demographic sample characteristics and overview of excluded participants in the face recognition condition.

			Age		Gender			*n* excluded based on	
					
Study	Pre-registered	Final *N*	*Median*	*SD*	male	female	other	Ethnicity	Performance below chance
Pilot	No	44	38.5	12.15	22	22	0	15	14
Study 1	Yes	66	34	10.35	35	30	1	21	1
Study 2	No	62	35	11.45	31	31	0	27	1
Study 3	No	73	35	14.21	28	45	0	29	0
Study 4	Yes	130	35	11.44	51	79	0	47	4
Study 5	Yes	127	36	13.00	58	68	1	40	6
Study 6	Yes	432	34	12.64	208	217	7	3	13


*Exclusion criteria were pre-specified in the pre-registrations.*

#### Materials

##### Names

We pretested 180 male and 173 female given names for their perceived typicality for Black versus White Americans on a 7-point scale (1 = “Very common for Whites,” 4 = “Both,” 7 = “Very common for Blacks,” or “neither”; see Supplementary Material for details). The names were identified from previous studies (Daniel and Daniel, 1998; Greenwald et al., 1998; Bertrand and Mullainathan, 2004; Levitt and Dubner, 2005; Cotton et al., 2008; Tzioumis, 2015) and from data of the NYC Health Department released in 2007^2^. We selected 40 male and 40 female typical White names and 20 male and 20 female typical Black names, as indicated by the pretest-ratings. Male Black names were perceived as significantly more typical for Black Americans (*M* = 6.29, *SD* = 0.14) than male White names (*M* = 2.16, *SD* = 0.19), *t*(58) = 86.058, *p* < 0.001, *d* = 23.57, 95% *CI*[19.14; 27.79]. Similarly, female Black names were perceived as significantly more typical for Black Americans (*M* = 6.56, *SD* = 0.10) than female White names (*M* = 1.85, *SD* = 0.16), *t*(58) = 120.900, *p* < 0.001, *d* = 33.11, 95% *CI*[26.92; 39.02].

Additionally, we compared the overall frequency of selected Black and White names in the United States society based on data from the Social Security Administration^3^ from the years between 1975 and 1995, which contains a list of 48,453 names with an aggregated frequency of 74,180,519 individuals. We restricted the analysis of name frequencies to this time period, because we assumed that the age group of people born between the years 1975 and 1995 would be most representative for the targeted participant group. Selected male Black names (*M* = 8,846, *SD* = 6,948) were significantly less frequent than selected male White names (*M* = 176,616, *SD* = 191,978), *t*(58) = 3.890, *p* < 0.001, *d* = 1.07; 95% *CI*[0.49; 1.63]. Similarly, selected female Black names (*M* = 8,676, *SD* = 11,305) were significantly less frequent than selected female White names (*M* = 147,599, *SD* = 147,129), *t*(58) = 4.199, *p* < 0.001, *d* = 1.14, 95% *CI*[0.57; 1.72]. In order to prevent that expected effects of ethnic typicality on recognition of Black faces are attributable to their low frequency, we additionally selected further control names—20 male and 20 female—whose frequencies matched the selected Black names (*M*_male_ = 9,144, *SD* = 9,563), *t*(38) = 0.113, *p* = 0.911, *d* = 0.04, 95% *CI*[-0.58; 0.66]; (*M*_female_ = 8,648, *SD* = 11,231), *t*(38) = 0.008, *p* = 0.994, *d* = 0.00, 95% *CI*[-0.62; 0.62]), while being perceived as neither typical Black nor White (*M*_male_ = 3.45, *SD* = 0.69 and *M*_female_ = 3.50, *SD* = 1.24), see Supplementary Tables S5, S7.

##### Faces

We selected photographs of 40 male Black faces, 40 female Black faces, 40 male White faces, and 40 female White faces from the Chicago Face Database (CFD; Ma et al., 2015). All images were presented in a size of 550 × 351 pixels.

#### Design

The learning phase followed a 2 (Face Ethnicity: Black versus White) by 3 (Name Condition: White versus Black/White versus Infrequent) nested design, with Face Ethnicity and Name Condition as within-participant factors. The factor Name Condition was nested under the factor Face Ethnicity in the following way: Black faces were combined with White names or Black names; White faces were combined with White names or infrequent names. After the learning phase, participants were randomly assigned to complete either the face recognition test or the name recognition test.

#### Procedure

The online study was created with Qualtrics survey software and distributed via MTurk. The survey was advertised as a study on memory for faces and names. After giving initial consent, participants completed an instructional manipulation check (see Oppenheimer et al., 2009). They confirmed having read instructions carefully by clicking on an icon in the upper right corner of the screen instead of intuitively clicking on the ‘next’ button. For participants who failed the instructional manipulation check (*n*_Pilot_ = 55; *n*_Study 1_ = 179; *n*_Study 2_ = 185), the experiment was immediately terminated. All other participants were presented with a random sequence of 40 study faces, each paired with a name displayed centrally above the faces. Each face was presented for 5 s. In order to counter-balance name-assignment, we created four different stimulus-sets with fixed assignments of names and faces—see Supplementary Tables S6, S8. Each study-set consisted of 10 Black faces with typical Black names, 10 Black faces with typical White names, 10 White faces with typical White names, and 10 White faces with infrequent names. The sets were randomly assigned to participants.

After the study phase, participants were randomly assigned to one of two recognition tests. In the face test, they were presented with the 40 faces from the study phase intermixed with 40 new faces. In the name test, they were presented with the 40 names from the study phase intermixed with 40 new names. Participants’ task was to indicate via mouse click whether a face/name was old or new. Following the recognition test, participants in Study 1 and 2 completed a contact questionnaire (see Supplementary Material for details on procedure and results), provided demographic information (age, gender, and ethnicity), were fully debriefed about the purpose and design of the study and gave their consent. The study took approximately 12 min.

### Results

#### ORE in Face Recognition

To test for an overall ORE in face recognition, we computed signal detection parameter d′ [d′ = z(Hit)- z(FA)] separately for Black and White faces. Hit and False Alarm rates of 1 or 0 were replaced with a minimum value of 0.5/n or a maximum of (n-0.5)/n, with n being the total of ‘old’ or ‘new’ trials, respectively. Mean d′ values are depicted in **Table 2**. Planned one-tailed paired *t*-tests indicated that d′ values were significantly higher for White faces compared to Black faces in the Pilot study, *t*(43) = 4.528, *p* < 0.001, *d*_*z*_ = 0.68, 90% *CI*[0.40; ∞], in Study 1, *t*(65) = 5.250, *p* < 0.001, *d*_*z*_ = 0.65, 90% *CI*[0.42; ∞], and in Study 2, *t*(61) = 3.773, *p* < 0.001, *d*_*z*_ = 0.48, 90% *CI*[0.26; ∞]. Thus, we consistently found a classic ORE in d′ values across all three studies.

**Table 2:** Recognition performance for outgroup versus ingroup faces, measured via signal detection parameter d′, hit rates, and false alarm rates.

	d′		Hit rates		False alarm rates	
			
	Outgroup faces	Ingroup faces	Outgroup faces	Ingroup faces	Outgroup faces	Ingroup faces
Pilot	0.93 (0.75)	1.48 (0.95)	0.68 (0.19)	0.70 (0.18)	0.38 (0.24)	0.25 (0.19)
Study 1	1.00 (0.72)	1.50 (0.79)	0.66 (0.15)	0.68 (0.17)	0.32 (0.18)	0.21 (0.15)
Study 2	1.32 (0.79)	1.63 (0.97)	0.71 (0.16)	0.70 (0.17)	0.29 (0.20)	0.20 (0.17)
Study 3	1.30 (0.73)	1.66 (0.76)	0.67 (0.18)	0.70 (0.16)	0.26 (0.17)	0.18 (0.15)
Study 4	1.22 (0.69)	1.73 (0.86)	0.68 (0.16)	0.70 (0.16)	0.28 (0.18)	0.17 (0.15)
Study 5	1.41 (0.70)	1.86 (0.96)	0.68 (0.17)	0.74 (0.18)	0.23 (0.16)	0.18 (0.15)
Study 6	1.05 (0.69)	1.56 (0.82)	0.65 (0.17)	0.67 (0.17)	0.30 (0.18)	0.19 (0.15)


*In all studies, outgroup faces are Black and ingroup faces are White; only in Study 3, outgroup faces are Chinese.*

In subsequent exploratory analyses, we computed two two-sided paired *t*-tests to compare hit rates and false alarm rates for Black versus White faces. Mean hit and false alarm rates are depicted in **Table 2**. Analysis of hit rates did not reveal significant differences for Black versus White faces, neither in the Pilot study, *t*(43) = 0.986, *p* = 0.330, *d*_*z*_ = 0.15, 90% *CI*[-0.15; 0.44], nor in Study 1, *t*(65) = 1.143, *p* = 0.257, *d*_*z*_ = 0.14, 95% *CI*[-0.10; 0.38], nor in Study 2, *t*(61) = 0.471, *p* = 0.639, *d*_*z*_ = 0.06, 95% *CI*[-0.19; 0.31]. Conversely, analysis of false alarm rates showed that there were significantly more false alarms for Black faces compared to White faces in the Pilot study, *t*(43) = 5.132, *p* < 0.001, *d*_*z*_ = 0.77, 95% *CI*[0.43; 1.11], as well as in Study 1, *t*(65) = 5.616, *p* < 0.001, *d*_*z*_ = 0.69, 95% *CI*[0.42; 0.96], and in Study 2, *t*(61) = 3.840, *p* < 0.001, *d*_*z*_ = 0.49, 95% *CI*[0.22; 0.75]. This suggests that the ORE in d′ values was driven by higher false alarm rates rather than by lower hit rates for Black faces.

#### Effect of Name Typicality on the ORE in Face Recognition

To test the influence of names on recognition of Black faces, we compared hit rates for Black faces when previously paired with White names versus Black names. We used hit rates as dependent variable, because this analysis was restricted to ‘old’ faces; ‘New’ faces in the recognition test did not carry names, thus not allowing computation of d′. Mean hit rates for the four name/ethnicity combinations are illustrated in **Table 3**. Planned one-tailed paired *t*-tests indicated that hit rates were significantly higher for Black faces with White names compared to Black faces with Black names in the Pilot study, *t*(43) = 2.081, *p* = 0.021, *d*_*z*_ = 0.31, 90% *CI*[0.06; ∞], in Study 1, *t*(65) = 1.924, *p* = 0.029, *d*_*z*_ = 0.24, 90% *CI*[0.03; ∞], and in Study 2, *t*(61) = 1.873, *p* = 0.033, *d*_*z*_ = 0.24, 90% *CI*[0.03; ∞]. Thus, results consistently showed that Black faces were better recognized when paired with White names than with Black names.

**Table 3:** Hit rates (*SD*) for recognition of outgroup and ingroup faces as a function of their combination with outgroup names, ingroup names, or infrequent names.

	Outgroup faces/Outgroup names	Outgroup faces/Ingroup names	Ingroup faces/Infrequent names	Ingroup faces/Ingroup names
Pilot	0.65 (0.21)	0.71 (0.23)	0.72 (0.20)	0.69 (0.22)
Study 1	0.64 (0.18)	0.68 (0.20)	0.70 (0.20)	0.67 (0.18)
Study 2	0.69 (0.20)	0.74 (0.18)	0.69 (0.19)	0.72 (0.20)
Study 3	0.65 (0.22)	0.69 (0.21)	0.68 (0.17)	0.71 (0.20)
Study 4	0.69 (0.20)	0.68 (0.18)	0.71 (0.19)	0.69 (0.19)
Study 5	0.67 (0.20)	0.69 (0.20)	0.71 (0.21)	0.76 (0.19)
Study 6	0.65 (0.20)	0.65 (0.20)	0.68 (0.19)	0.66 (0.20)


*Statistical tests for comparisons of the other face/name conditions are reported in the Supplementary Table S1. In all studies, outgroup faces are Black and ingroup faces are White; only in Study 3, outgroup faces are Chinese.*

### Discussion

We conducted three studies investigating the influence of name typicality on the ORE in face recognition. In addition to a classic ORE, we observed a recognition advantage for Black faces with White names over Black faces with Black names. These results provide initial evidence that the ORE can be modulated by typicality of given names. In order to investigate the generalizability of the effect, we conducted a conceptual replication study, in which we included faces and names of a different outgroup.

## Study 3

Study 3 aimed at conceptually replicating effects from Studies 1 and 2 with Chinese and White faces and names. It is very common that Chinese Americans adopt English given names in addition to their Chinese names (Louie, 1998). This study investigates if Chinese versus English names influence memory for Chinese faces in White American participants.

### Method

#### Participants

Participants were again recruited via MTurk with the same selection criteria, same payment, and same exclusion criteria as in Studies 1 and 2. Our rationale for sample size estimation was identical to Studies 1 and 2. Final sample sizes, number of excluded participants, and demographic information are reported in **Table 1**. For additional 202 participants who failed the instructional manipulation check, the experiment was immediately terminated.

#### Material

##### Names

We pretested 49 male White given names and 50 Chinese given names for their perceived typicality for Chinese versus White Americans on a 7-point scale (1 = “Very common for Whites,” 4 = “Both,” 7 = “Very common for Chinese,” or “neither”; see Supplementary Material for details). White names were selected from the previous pretest-set; Chinese names were selected from the Social Security Administration, with the restriction that the male/female ratio was at least 80%. Based on the pretest, we selected the 40 most typical White names and the 20 most typical Chinese names. Chinese names were perceived as significantly more typical for Chinese Americans (*M* = 6.74, *SD* = 0.16) than male White names (*M* = 1.32, *SD* = 0.10), *t*(26.6) = 137.130, *p* < 0.001, *d* = 37.55, 95% *CI*[30.54; 44.25].

We compared the overall frequency of the selected Chinese and White names in the United States society based on data from the Social Security Administration from the years between 1975 and 1995. Selected male Chinese names (*M* = 228, *SD* = 161) were significantly less frequent than selected male White names (*M* = 183,682, *SD* = 201,744), *t*(39) = 5.751, *p* < 0.001, *d* = 5.75, 95% *CI*[4.57; 6.91]. In order to control that expected effects of ethnic typicality are not attributable to their low frequency, we additionally selected 20 control names whose frequencies matched the selected Chinese names (*M* = 238, *SD* = 163, *t*(36.9) = 0.180, *p* = 0.858, *d* = 0.06, 95% *CI*[-0.56; 0.67]; see Supplementary Tables S9, S10).

##### Faces

We selected photographs of 40 male White faces, 30 male Asian American faces from the CFD and 10 male Chinese faces from the Chinese University of Hong Kong Face Sketch database (CUFS; Wang and Tang, 2009), for which we adjusted the color of backgrounds and clothes to resemble the images from the CFD. All images were presented on screen in a size of 550 × 351 pixels.

#### Design and Procedure

Design and procedure were identical to Studies 1 and 2. In order to counter-balance name assignment, we again created four different stimulus-sets with fixed assignments of names and faces—see Supplementary Table S11.

### Results

#### ORE in Face Recognition

To test for an overall other-race effect in face recognition, we compared d′ values for Black and Chinese faces—see **Table 2**. A planned one-tailed paired *t*-test indicated a classic ORE: d′ values were significantly higher for White faces compared to Chinese faces, *t*(72) = 4.011, *p* < 0.001, *d*_*z*_ = 0.47, 90% *CI*[0.26; ∞].

Exploratory two-sided paired *t*-tests demonstrated that hit rates did not significantly differ for Chinese faces compared to White faces, *t*(72) = 1.254, *p* = 0.214, *d*_*z*_ = 0.14, 95% *CI*[-0.08; 0.38], while there were significantly more false alarms for Chinese faces compared to White faces, *t*(72) = 4.201, *p* < 0.001, *d*_*z*_ = 0.49, 95% *CI*[0.25; 0.73]—see **Table 2**. This pattern replicates effects from previous studies, suggesting again that the ORE in d’ values was driven by higher false alarm rates for Chinese faces instead of higher hit rates for White faces.

#### Effect of Name Typicality on the ORE in Face Recognition

To test the influence of names on recognition of Chinese faces, we compared hit rates for Chinese faces when previously paired with White names versus Chinese names—see **Table 3**. Planned one-tailed paired *t*-test indicated that hit rates were significantly higher for Chinese faces with White names compared to Chinese faces with Chinese names, *t*(72) = 1.777, *p* = 0.040, *d*_*z*_ = 0.21, 90% *CI*[0.01; ∞].

### Discussion

Study 3 aimed at replicating effects from previous studies with a different outgroup. Similar to previous studies, we observed a recognition advantage for Chinese faces with White names compared to Chinese faces with Chinese names. These results provide further evidence that the ORE can be modulated by typicality of names. However, it should be noted that the effect sizes of all four studies were only small to moderate (*d*_*z*_ = 0.21–0.31). Given that the sample sizes were quite small to detect such small effects—yielding an average test power of 1 – β = 0.60—we conducted two further studies in order to replicate the effect with two larger samples.

## Studies 4 and 5

Studies 4 and 5 aimed at replicating the effect of Black versus White names on recognition of Black faces from Studies 1 and 2 with larger sample sizes. Data collection procedures, sample sizes, and analysis plans of both studies were pre-registered on OSF.

### Method

#### Participants

Participants were recruited via MTurk with the same selection criteria, same payment, and same exclusion criteria as in Studies 1–3. Sample sizes were estimated via *a priori* power analyses conducted with G^∗^Power 3.1 for a one-tailed paired *T*-tests with 1-β = 0.85, α = 0.05, based on the average effect size estimate of Studies 1 and 2 (*d*_*z*_ = 0.24). Final sample sizes, number of excluded participants, and demographic information are reported in **Table 1**. For participants who failed the instructional manipulation check (*n*_Study 4_ = 422; *n*_Study 5_ = 444), the experiment was immediately terminated.

#### Materials, Design, and Procedure

Materials, design, and procedure were identical to Studies 1and 2, except that participants did not complete the contact questionnaire. Study 4 included the same stimulus set as Study 1; Study 5 included the same stimulus set as Study 2.

### Results

#### ORE in Face Recognition

To test for an overall ORE in face recognition, we again compared d′ values for Black and White faces—see **Table 2**. Planned one-tailed paired *t*-tests indicated that d’ values were significantly higher for White faces compared to Black faces in Study 4, *t*(129) = 7.095, *p* < 0.001, *d*_*z*_ = 0.62, 90% *CI*[0.46; ∞], as well as in Study 5, *t*(126) = 6.395, *p* < 0.001, *d*_*z*_ = 0.57, 90% *CI*[0.41; ∞], thus replicating the classic ORE.

An exploratory two-sided paired *t*-test of hit rates did not reveal significant differences for Black versus White faces in Study 4, *t*(129) = 0.973, *p* = 0.332, *d*_*z*_ = 0.09, 95% *CI*[-0.08; 0.26]. However, in Study 5, hit rates were higher for White faces compared to Black faces, *t*(126) = 3.564, *p* < 0.001, *d*_*z*_ = 0.31, 95% *CI*[0.13; 0.49]. Analysis of false alarm rates showed that there were significantly more false alarms for Black faces compared to White faces, in Study 4, *t*(129) = 8.132, *p* < 0.001, *d*_*z*_ = 0.71, 95% *CI*[0.51; 0.90], as well as in Study 5, *t*(126) = 3.525, *p* < 0.001, *d*_*z*_ = 0.31, 95% *CI*[0.13; 0.49]. These results broadly replicate results from the previous studies, except that there was a significant effect in hit rates in Study 5.

#### Effect of Name Typicality on the ORE in Face Recognition

To test the influence of names on recognition of Black faces, we again compared hit rates for Black faces when previously paired with White names versus Black names—see **Table 3**. Planned one-tailed paired *t*-test indicated that hit rates did *not* differ for Black faces with White names compared to Black faces with Black names, neither in Study 4, *t*(129) = -0.893, *p* = 0.813, *d*_*z*_ = -0.08, 90% *CI*[-∞; 0.06], nor in Study 5, *t*(126) = 1.026, *p* = 0.154, *d*_*z*_ = 0.09, 90% *CI*[-∞; 0.24]. Thus, results of the previous studies did not replicate in Study 4 and 5.

### Discussion

Studies 4 and 5 aimed at replicating effects from Studies 1 and 2 with larger samples. Similar to the previous studies, we observed an overall other-race effect, indicating better memory for White faces compared to Black faces. Again, this effect was more strongly driven by higher false alarm rates for Black faces, whereas the effect in hit rates was not significant. However, while Studies 1 and 2 found an influence of name typicality on recognition of outgroup faces, this effect did not replicate in Studies 4 and 5. As has recently been argued, finding such an inconsistent pattern of results is not unlikely (Lakens and Etz, 2017). We therefore chose a meta-analytic approach to summarize effects across all five studies and to achieve a more precise effect size estimate (cf. Goh et al., 2016). Results of this meta-analysis showed that the overall effect of name typicality was still significant, but it was very small (*d*_*z*_ = 0.14). Given that the two larger studies did not show any effects, we conducted another high-powered study in order to accumulate more evidence on whether the effect of name typicality is reliable.

## Study 6

In order to address the inconclusive pattern of results from previous studies, Study 6 aimed at replicating the effect in a larger sample. We chose to replicate the effect for Black versus White male faces (Pilot, Studies 1 and 4), because this effect has shown the highest inconsistency. Different to the previous studies, participants were recruited via the online platform Prolific, in order to safeguard against recently discussed problems of MTurk (e.g., Peer et al., 2017), which may have contributed to the non-replications in Studies 4 and 5. Data collection procedure, sample size, and analysis plan were again pre-registered on OSF.

### Method

#### Participants

A targeted sample size of *N* = 431 was estimated via an *a priori* power analysis conducted with G^∗^Power 3.1 for a one-tailed paired *T*-test with 1-β = 0.80, α = 0.05, for a small effect size of *d*_*z*_ = 0.12. We obtained the effect size estimate via an internal meta-analysis including all previous studies with Black faces (i.e., all studies, excluding Study 3). Participants were recruited via Prolific with the selection criteria that (a) their nationality, their country of birth, and their current country of residence was the United States, (b) their ethnicity was White/Caucasian, and (c) their approval rate was not lower than 90%. Participants received £1.25 British Pound for their participation. Final sample size, number of excluded participants, and demographic information are reported in **Table 1**. For additional 679 participants who failed the instructional manipulation check the experiment was immediately terminated.

#### Materials, Design, and Procedure

Materials, design, and procedure were identical to Studies 1 and 4.

### Results

#### ORE in Face Recognition

We again compared d’ values for Black and White faces—see **Table 2**. Planned one-tailed paired *t*-tests indicated that d’ values were significantly higher for White faces compared to Black faces, *t*(431) = 15.232, *p* < 0.001, *d*_*z*_ = 0.73, 90% *CI*[0.64; ∞], again replicating the classic ORE.

Exploratory analysis of hit rates in a two-sided paired *t*-test showed that hit rates were significantly higher for White faces compared to Black faces, *t*(431) = 2.777, *p* = 0.006, *d*_*z*_ = 0.13, 95% *CI*[0.04; 0.23]. Similarly, analysis of false alarm rates showed that there were significantly more false alarms for Black faces compared to White faces, *t*(431) = 14.080, *p* < 0.001, *d*_*z*_ = 0.68, 95% *CI*[0.57; 0.78]. These effects replicate the general pattern of results from the previous studies: Even though the effect was significant in hit rates in this study, the effect size was larger in false alarm rates, suggesting that the ORE in d′ values was again largely driven by false alarms.

#### Effect of Name Typicality on the ORE in Face Recognition

To test the influence of names on recognition of Black faces, we again compared hit rates for Black faces when previously paired with White names versus Black names—see **Table 3**. Planned one-tailed paired *t*-test indicated that hit rates did *not* differ for Black faces with White names compared to Black faces with Black names, *t*(431) = 0.505, *p* = 0.307, *d*_*z*_ = 0.02, 90% *CI*[-∞; 0.10]. Thus, the result of Study 6 replicates findings from Studies 4 and 5, suggesting that recognition of Black faces was not influenced by name typicality.

### Discussion

Study 6 is a high powered close replication of Studies 1 and 4. Contrary to our expectations, we did not find a significant effect of name typicality on recognition of Black faces: While we found an overall other-race effect, we did not observe a difference in memory performance for Black faces with White names compared to Black faces with Black names. Again, the other-race effect was mainly driven by increased false alarm rates for Black faces, whereas the difference in hit rates for Black versus White was only small.

## Mini Meta-Analysis

**FIGURE 1:**
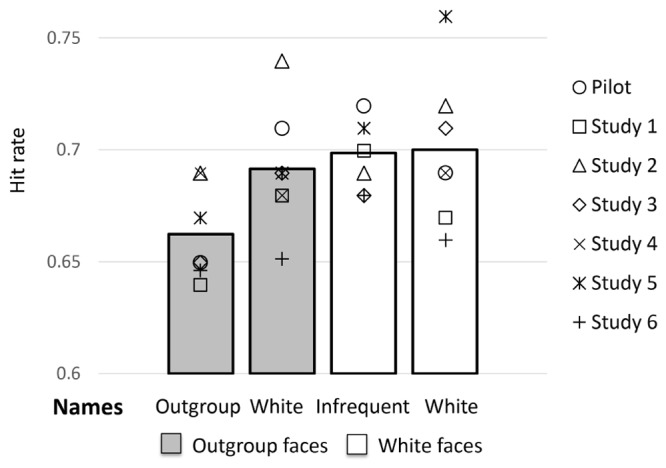
Hit rates for Outgroup and White faces with Outgroup versus White names. Gray and white bars represent hit rates averaged across all studies; the means of the single studies were weighted with the inverse variance of the effect size before averaging. Symbols represent average hit rates of the single studies.

The effect of name typicality on recognition of outgroup faces has been observed in four out of seven studies. Particularly, the high-powered studies failed to replicate the effect. It has recently been argued that finding an inconsistent pattern of results is not necessarily uncommon (Lakens and Etz, 2017). We therefore chose a meta-analytic approach to summarize effects across all seven studies, in order to achieve a more precise effect size estimate (Goh et al., 2016). Weighted means of the seven studies are depicted in **Figure 1**.

We used both random-effects and fixed-effects models to analyze the influence of name typicality on recognition of outgroup faces. We used standardized mean differences for dependent measurement designs (Cohen’s *d*_*z*_) as effect size, computed via the formula $d=t/\sqrt{N}$ (Rosenthal, 1991). Effect sizes were weighted by the inverse variance of the effect size, estimated via the formula ((1/*N*)+((d^∧^2)/(2^∗^N)))^∗^2^∗^(1 - *r*), as suggested by Borenstein et al. (2009). The meta-analyses were computed using the R package ‘metafor’ (Viechtbauer, 2010).

**FIGURE 2:**
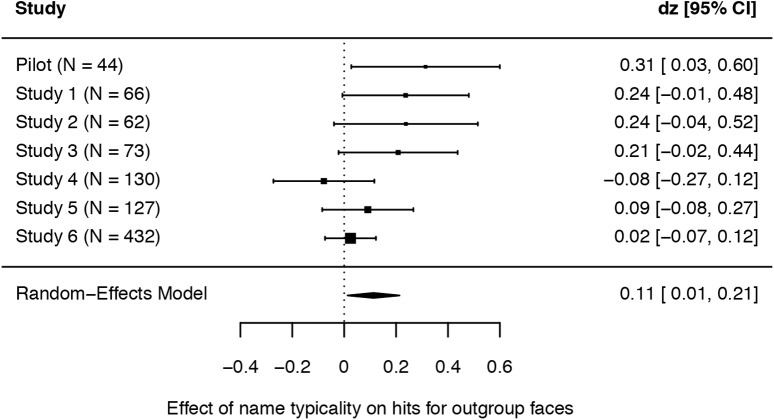
Forest plot for the comparison of hit rates for outgroup faces with outgroup versus White names in the random-effects model. Effect sizes are weighted with the inverse variance of the effect size. Note that, contrary to the one-sided *t*-tests reported for the single studies, the forest plot displays 95% confidence intervals.

The random-effects model revealed a significant small effect of name typicality on hit rates for Black faces, *d*_*z*_ = 0.11, 95% *CI*[0.01; 0.21], *p* = 0.027, see **Figure 2**. Similarly, the fixed-effects model revealed a significant small effect of name typicality on hit rates for Black faces, *d*_*z*_ = 0.08, 95% *CI*[0.01; 0.15], *p* = 0.017. The test for heterogeneity was not significant, *Q*(6) = 10.394, *I*^2^ = 43.96%, *p* = 0.109.

## General Discussion

We conducted seven studies investigating if the ORE is reduced for Black or Chinese outgroup faces when paired with White names compared to Black or Chinese names. Different lines of reasoning made it plausible for us to predict such an effect: First, we argued that White names may contradict with participants’ initial categorization, thus, leading to more individuated processing (see Fiske and Neuberg, 1990). Second, we argued that White names may reduce perceived prototypicality of the faces, thus, serving as an additional racial marker. This may alter the categorization process, which in turn may influence face recognition (see Maclin and Malpass, 2001). Third, we argued that Black individuals with White names may be perceived to have a higher social status (see Fryer and Levitt, 2004; Cotton et al., 2008), thus, increasing motivation to individuate (see Shriver et al., 2008).

Contrary to our expectations, we observed an inconsistent effect of name typicality on outgroup face recognition. While we found significant effects in the first four studies, these effects did not reproduce in three high-powered replications studies. Finding a pattern of mixed results is not necessarily uncommon (Lakens and Etz, 2017); therefore, we summarized effects in an internal meta-analysis. However, the meta-analysis should be interpreted with caution, given that particularly the high-powered and (all-but-one) pre-registered studies did not show significant effects. While the meta-analysis accounts for differences in test power (i.e., effect sizes were weighted with inverse variances, which are related to sample sizes), the analysis does not take into account if a study was preregistered. If we included only pre-registered studies in the meta-analysis, this would lead to the conclusion that there is no effect of name typicality on the ORE. However, we decided to include all studies because (a) all studies employed identical data collection and analysis procedures, regardless of pre-registration, and (b) we did not want to produce a file drawer by including only a subset of studies. Having the above-mentioned restrictions in mind, results of the meta-analysis demonstrated a very small (*d*_*z*_ = 0.11), but significant effect. Note that the effect size estimation is as precise as it can get, because we included all the data we have collected, and thus, no publication bias can have affected the results.

While the effect of name typicality was inconsistent across the studies, we consistently observed a general ORE: A recognition advantage of White faces compared to outgroup faces in all seven studies. This replicates findings from previous studies, showing that the ORE is a very robust phenomenon (see Meissner and Brigham, 2001). Taking a closer look, the ORE was mainly driven by more incorrect responses to *new* outgroup faces (i.e., more false alarms), rather than by incorrect responses to *old* outgroup faces (i.e., hits). This particular pattern of recognition performance may partly explain why we did not find a consistent effect of name typicality on outgroup faces. Specifically, our procedure allowed analysis of name-typicality effects only in responses to old faces (hit rates), because the recognition test included only faces without any names. Conversely, new faces were never paired with names, thus, not allowing to test the influence of names on false alarm rates. Given that the ORE was mainly driven by false alarm rates, hit rates may be a less sensitive measure for an effect of name typicality as compared to false alarms. This may be one reason why the effects were inconsistent. Future studies may use altered experimental procedures allowing additional analyses of false alarms (e.g., presenting names in the learning phase and in the recognition phase). Alternatively, future studies may employ a forced-choice format, presenting participants with several familiar and new faces, asking to decide ‘Who is Emily?’. Furthermore, future studies may benefit from employing a fully crossed design, using the identical names for both conditions (i.e., infrequent names with Black faces, as well as Black names with White faces). Also, future research may explore if and to what extent the observed effects are specific for face memory or would arise for any other person memory as well. These approaches may further help quantifying the impact of name typicality on face recognition.

Assuming that future research may find further evidence of a small effect of name typicality on outgroup face recognition, one may still argue that this effect is too small to have any practical relevance. In fact, results of an equivalence test (see Lakens, 2017) computed for the present data showed, that, if an effect exists, it has to be smaller than the conventionally defined small effect size of *d*_*z*_ = 0.20 (*Z* = -1.704, *p* = 0.044). However, we think that small effects may still be of theoretical relevance. For example, even if small, an effect of name typicality would be a further indicator for social moderation effects on the ORE, which is often ignored in expertise-based theories of face processing perspectives. These theories explain the ORE mainly with effects of mere (lack of) exposure and contact and thus insufficient proficiency in individuating outgroup faces (e.g., Rhodes et al., 1989; Valentine, 2001; Tanaka et al., 2004; Hancock and Rhodes, 2008). In this regard, even small effects can be informative. Furthermore, small effects may still have (severe) real-live consequences, either because they can affect many people simultaneously or because they can affect single persons repeatedly (e.g., Greenwald et al., 2015). Going back to our introductory example, among the many everyday experiences in which face recognition plays an important role, a minority member with a typical name of the minority group may still make more frequent experiences of not being recognized than a minority member with a White name, which may have consequences in many areas of everyday life.

In summary, we present initial evidence of an influence of name typicality on recognition of outgroup faces. However, the effect should be interpreted with caution, given that the effect has not been robustly found and the effect size is very small. Nonetheless, for the above-mentioned reasons, we do think that the current findings inspire further research on this phenomenon (i.e., with altered methodology), which may be fruitful to better understand the underlying processes and social moderators of the ORE, to increase ecological validity to ORE research, as well as help understanding the real-live relevance of the effect on minority group members.

## Ethics Statement

This study was carried out in accordance with the recommendations of the Human Research Participant guidelines of the American Psychological Association with written informed consent from all participants. At the time of data collection, ethical approval was not legally required for this kind of study.

## Author Contributions

MS organized preparation of stimulus material and data collection, performed the statistical analysis, and wrote the first draft of the manuscript. JD wrote sections of the manuscript. Both authors contributed to conception and design of the studies, manuscript revision and read and approved the submitted version.

## Conflict of Interest Statement

The authors declare that the research was conducted in the absence of any commercial or financial relationships that could be construed as a potential conflict of interest.

## Footnotes

1 Preregistrations, experimental procedures, raw data files, and analysis scripts can be downloaded from https://osf.io/g5ruy/

2 Retrieved from http://www.nyc.gov/html/doh/downloads/pdf/public/press07/pr085-07-babynames.pdf

3 Retrieved from https://www.ssa.gov/oact/babynames/names.zip
