# Supplementary material for: Recognizing Emily and Latisha: Inconsistent Effects of Name Stereotypicality on the Other-Race Effect
Source: Front Psychol. 2018 Apr 13;9:486. doi: 10.3389/fpsyg.2018.00486 (PMC5908905; doi:10.3389/fpsyg.2018.00486)
Supplement: Supplementary file 1 [file Data_Sheet_1.DOCX]

Supplementary Material

Recognizing Emily and Latisha: Inconsistent Effects of Name Stereotypicality on the Other-Race Effect

Marleen Stelter*, Juliane Degner

*** Correspondence:** Marleen Stelter: [Marleen.Stelter@uni-hamburg.de](mailto:Marleen.Stelter@uni-hamburg.de)

# Contact questionnaire

The contact questionnaire employed in Studies 1, 2, 3, and 6 consisted of 17 statements regarding participants’ amount of contact with White Americans and African Americans (Chinese Americans in Study 3) in their daily lives. Participants rated the statements on 6-point scales (1 = “Very strongly disagree”; 6 = “Very strongly agree”). Fifteen items were adopted from (Hancock and Rhodes 2008); we added two further statements asking for indirect contact via television and movies (“I watch a lot of television or movies with White American actors.”; “I watch a lot of television or movies with Black or African American (Chinese American) actors.”). Internal consistencies of the contact questionnaire were quite good, ranging from α = .75 - .84. Contact with Black Americans was not related to the ORE in face recognition, neither in Study 1, *r*(64) = -.018, *p* = .889, nor in Study 2, *r*(60) = .043, *p* = .741, nor in Study 6, *r*(60) = -.009, *p* = . 847. Similarly, contact with Chinese Americans was not correlated with the ORE in face recognition Study 3, *r*(71)*=*-.177, *p* = .135.

# Supplementary Tables

Table S1

*Exploratory comparison of hit rates for the different face/name conditions.*

|  | Outgroup faces/outgroup names  vs. White faces/White names | | | |  | | Outgroup faces/White names  vs. White faces/White names | | | | |  | | White faces/White names  vs. White faces/Infrequent names | | | |
| --- | --- | --- | --- | --- | --- | --- | --- | --- | --- | --- | --- | --- | --- | --- | --- | --- | --- |
|  | *t* | *df* | *p* | d_z_ [95% CI] | |  | | *t* | *df* | *p* | d_z_ [95% CI] | |  | *t* | *df* | *p* | d_z_ [95% CI] |
| Pilot | -1.058 | 43 | .296 | -0.16 [-0.45; 0.14] | |  | | 0.659 | 43 | .513 | 0.09 [-0.20; 0.39] | |  | -1.112 | 43 | .272 | -0.17 [-0.46; 0.13] |
| Study 1 | -1.144 | 65 | .257 | -0.14 [-0.38; 0.10] | |  | | 0.474 | 65 | .637 | 0.06 [-0.18; 0.30] | |  | -1.229 | 65 | .224 | -0.15 [-0.39; 0.09] |
| Study 2 | -1.000 | 61 | .321 | -0.13 [-0.37; 0.12] | |  | | 0.950 | 61 | .346 | 0.12 [-0.13; 0.37] | |  | 1.005 | 61 | .319 | 0.13 [-0.12; 0.38] |
| Study 3 | -2.244 | 72 | .028 | -0.26 [-0.50; -0.03] | |  | | -0.655 | 72 | .515 | -0.08 [-0.31; 0.15] | |  | 1.187 | 72 | .239 | 0.13 [-0.09; 0.37] |
| Study 4 | 0.037 | 129 | .971 | 0.00 [-0.17; 0.18] | |  | | -0.828 | 129 | .409 | -0.07 [-0.25; 0.10] | |  | -0.804 | 129 | .423 | -0.07 [-0.24; 0.10] |
| Study 5 | -4.849 | 126 | <.001 | -0.43 [-0.61; 0.25] | |  | | -3.655 | 126 | <.001 | -0.32 [-0.50; -0.16] | |  | 3.054 | 126 | .003 | 0.27 [0.09; 0.45] |
| Study 6 | -1.175 | 431 | .241 | -0.06 [-0.15; 0.04] | |  | | -0.707 | 431 | .480 | -0.03 [-0.13; 0.06] | |  | -2.420 | 431 | .016 | -0.12 [-0.21; -0.02] |

*Note.* Descriptive statistics are reported in *Table 3* of the main paper. In all studies, outgroup faces are Black and ingroup faces are White; only in Study 3, outgroup faces are Chinese.

Table S2

*Demographic sample characteristics and overview of excluded participants in the name recognition condition. Exclusion criteria were pre-specified in the pre-registrations.*

|  |  |  | Age | |  | Gender | | |  | *n* excluded based on | |
| --- | --- | --- | --- | --- | --- | --- | --- | --- | --- | --- | --- |
| Study | Pre-registered | Final *N* | *Median* | *SD* |  | male | female | other |  | Ethnicity | Performance below chance |
| Pilot | no | 51 | 39 | 11.84 |  | 17 | 34 | - |  | 21 | 7 |
| Study 1 | yes | 62 | 34 | 9.88 |  | 26 | 36 | - |  | 25 | 2 |
| Study 2 | no | 64 | 36.5 | 12.41 |  | 25 | 39 | - |  | 25 | 3 |
| Study 3 | no | 74 | 37 | 12.91 |  | 42 | 31 | 1 |  | 24 | 2 |
| Study 4 | yes | 127 | 35 | 11.39 |  | 57 | 70 | - |  | 39 | 2 |
| Study 5 | yes | 133 | 37 | 12.17 |  | 45 | 87 | 1 |  | 51 | 2 |

Table S3

*Descriptive and Inference Statistics for Name Recognition Performances.*

|  | *d'* | | |  | | Comparison:  Outgroup vs. White names^1^ | | |  | Comparison:  Outgroup vs. Infrequent names^2^ | | | |
| --- | --- | --- | --- | --- | --- | --- | --- | --- | --- | --- | --- | --- | --- |
|  | Outgroup names | Infrequent  names | White names |  | *t* | *df* | *p* | *d_z_* [90 % CI] |  | *t* | *df* | *p* | *d_z_* [95 % CI] |
| Pilot | 1.33 (1.00) | 1.65 (1.03) | 1.21 (0.83) |  | 1.009 | 50 | .841 | 0.14 [0.09; ∞] |  | 2.477 | 50 | .017 | 0.35 [0.06; 0.63] |
| Study 1 | 1.85 (0.90) | 2.18 (1.00) | 1.52 (0.87) |  | 3.279 | 61 | .999 | 0.42 [0.20; ∞] |  | 3.075 | 61 | .003 | 0.39 [0.13; 0.65] |
| Study 2 | 1.19 (0.70) | 2.12 (0.95) | 1.51 (0.81) |  | -2.863 | 63 | .003 | -0.36 [-∞; -0.14] |  | -7.872 | 63 | <.001 | -0.98 [-1.28; -0.68] |
| Study 3 | 1.32 (0.82) | 2.22 (0.93) | 1.43 (0.71) |  | -1.272 | 73 | .104 | -0.15 [-∞; 0.04] |  | -7.611 | 73 | <.001 | -0.88 [-1.15; -0.61] |
| Study 4 | 1.97 (0.90) | 2.33 (0.92) | 1.60 (0.73) |  | 4.560 | 126 | <.001 | 0.40 [0.25; ∞] |  | 4.392 | 126 | <.001 | 0.39 [0.20; 0.57] |
| Study 5 | 1.44 (0.68) | 2.28 (0.97) | 1.52 (0.76) |  | -1.083 | 132 | .140 | -0.09 [-∞; 0.05] |  | -9.309 | 132 | <.001 | -0.81 [-1.00; -0.61] |

Note. In all studies, outgroup names are typical Black names; only in Study 3, outgroup names are typical Chinese names. ^1^In accordance with the preregistrations (Hypothesis 3), this comparison was tested one-sided in the direction that recognition of outgroup names would be worse than recognition of White names. ^2^The comparison of recognition of outgroup vs. infrequent names was exploratory and was thus tested two-sided.

Table S4

*Hit rates (SD) for recognition of Black, White, and infrequent names depending on combination with Black or White faces.*

|  | Hit rates | | | |  | Comparison:  White names / White faces vs.  White names / Outgroup faces^1^ | | | |
| --- | --- | --- | --- | --- | --- | --- | --- | --- | --- |
|  | Outgroup names/  Outgroup faces | White names/  Outgroup faces | Infrequent names /  White faces | White names/  White faces |  | *t* | *df* | *p* | *d_z_* [90 % CI] |
| Pilot | 0.65 (0.21) | 0.71 (0.23) | 0.72 (0.20) | 0.69 (0.22) |  | -0.258 | 50 | .798 | -0.04 [-∞; 0.24] |
| Study 1 | 0.84 (0.15) | 0.62 (0.21) | 0.71 (0.21) | 0.68 (0.22) |  | 2.070 | 61 | .021 | 0.26 [0.05; ∞] |
| Study 2 | 0.83 (0.15) | 0.68 (0.21) | 0.72 (0.17) | 0.66 (0.19) |  | -0.637 | 64 | .737 | -0.08 [-∞; 0.12] |
| Study 3 | 0.78 (0.19) | 0.63 (0.19) | 0.81 (0.16) | 0.64 (0.21) |  | 0.591 | 73 | .278 | 0.07 [-0.26; ∞] |
| Study 4 | 0.87 (0.13) | 0.67 (0.20) | 0.75 (0.17) | 0.68 (0.20) |  | 0.947 | 126 | .172 | 0.08 [-0.06; ∞] |
| Study 5 | 0.87 (0.13) | 0.64 (0.20) | 0.72 (0.21) | 0.67 (0.21) |  | 0.375 | 132 | .354 | 0.03 [-0.11; 0.18] |

*Note.* ^1^ In accordance with the preregistrations (Hypothesis 4), this comparison was tested one-sided in the direction that recognition of White names paired with Outgroup faces would be worse than recognition of White names paired with White faces.

Table S5

*Results of the generalized linear mixed-effects model for comparison of hit rates for Black (Chinese) faces with Black (Chinese) vs. White names.*

| Study | Estimate | *SE* | *z* | *p* |
| --- | --- | --- | --- | --- |
| Pilot study | 0.407 | 0.196 | 2.074 | .038 |
| Study 1 | 0.204 | 0.155 | 1.314 | .189 |
| Study 2 | 0.264 | 0.229 | 1.154 | .249 |
| Study 3 | 0.278 | 0.133 | 2.093 | .036 |
| Study 4 | -0.207 | 0.123 | -1.683 | .093 |
| Study 5 | -0.016 | 0.140 | -0.115 | .909 |
| Study 6 | 0.021 | 0.067 | 0.311 | .756 |

*Note.* We used R (R Core Team, 2017) and lme4 (Bates, Maechler, Bolker, & Walker, 2015) to perform a generalized linear mixed-effects analysis of the relationship between name typicality and recognition of black faces, while treating participants and face stimuli as random factors (see Judd, Westfall, & Kenny, 2012). As fixed effects, we entered name typicality into the model. As random effects, we included intercepts for participants and faces, as well as by-participant and by-face random slopes for the effect of name typicality. Names were not included as random factors, because name-to-face assignments were fixed (see *Tables S7*, *S9*, and *S11*). P-values were obtained via Wald’s *z* statistics.

Table S6

List of male first names used in Pilot study and Studies 1, 4, and 6 with frequencies based on Social Security Data from 1975-1995 and descriptive statistics of stereotypicality ratings.

|  |  | | Stereotypicality | | | | | |
| --- | --- | --- | --- | --- | --- | --- | --- | --- |
|  | Frequency | | *N* | | *M* | *SD* | *N* (neither) | |
| Typical Black American Male First Names | | | | | | | | |
| Deandre | 12271 | 42 | | 6.33 | | 1.14 | 0 |  |
| Deion | 1616 | 38 | | 6.26 | | 1.03 | 3 |  |
| Demetrius | 16175 | 31 | | 6.00 | | 1.37 | 3 |  |
| Deshawn | 5832 | 41 | | 6.54 | | 0.81 | 1 |  |
| Donte | 7649 | 35 | | 6.17 | | 1.15 | 0 |  |
| Hakeem | 2185 | 28 | | 6.46 | | 1.11 | 7 |  |
| Jamal | 16815 | 41 | | 6.46 | | 0.98 | 1 |  |
| Jamel | 6114 | 35 | | 6.29 | | 1.27 | 0 |  |
| Jermaine | 23473 | 35 | | 6.29 | | 1.05 | 0 |  |
| Kareem | 6981 | 33 | | 6.42 | | 0.83 | 2 |  |
| Lamar | 11133 | 34 | | 6.38 | | 1.23 | 0 |  |
| Lamont | 8149 | 34 | | 6.29 | | 1.03 | 1 |  |
| Malik | 7740 | 39 | | 6.13 | | 1.22 | 3 |  |
| Marcellus | 1665 | 41 | | 6.12 | | 1.38 | 1 |  |
| Marquis | 11780 | 41 | | 6.17 | | 1.14 | 1 |  |
| Rasheed | 2188 | 30 | | 6.50 | | 0.90 | 5 |  |
| Tremayne | 2168 | 33 | | 6.36 | | 1.17 | 1 |  |
| Trevon | 3315 | 42 | | 6.19 | | 1.23 | 0 |  |
| Tyree | 4891 | 41 | | 6.22 | | 1.26 | 1 |  |
| Tyrone | 24779 | 42 | | 6.29 | | 1.11 | 0 |  |
| Alan | 52888 | 35 | | 2.51 | | 1.29 | 17 |  |
| Andrew | 537371 | 35 | | 2.14 | | 1.06 | 0 |  |
|  | Frequency | *N* | | *M* | | *SD* | *N* (neither) |  |
| Typical White American Male First Names | | | | | | | |  |
| Arthur | 34358 | | 34 | | 2.32 | 1.20 | 1 |  |
| Brad | 28519 | | 35 | | 1.77 | 1.00 | 0 |  |
| Bradley | 132494 | | 40 | | 2.20 | 1.49 | 0 |  |
| Brandon | 438807 | | 42 | | 2.31 | 1.32 | 0 |  |
| Brett | 77176 | | 34 | | 1.94 | 0.92 | 1 |  |
| Brian | 466816 | | 34 | | 2.15 | 1.13 | 0 |  |
| Cody | 173611 | | 42 | | 1.93 | 0.97 | 0 |  |
| Colin | 38121 | | 35 | | 2.40 | 1.31 | 0 |  |
| Connor | 31302 | | 41 | | 2.05 | 1.30 | 0 |  |
| Dennis | 63849 | | 35 | | 2.37 | 1.00 | 0 |  |
| Douglas | 83845 | | 34 | | 2.35 | 1.20 | 0 |  |
| Drew | 29457 | | 35 | | 2.34 | 1.26 | 0 |  |
| Dustin | 157079 | | 40 | | 2.10 | 1.13 | 0 |  |
| Dylan | 83202 | | 42 | | 2.07 | 1.16 | 0 |  |
| Edward | 125444 | | 34 | | 2.18 | 1.29 | 1 |  |
| Ethan | 44897 | | 42 | | 1.83 | 1.12 | 0 |  |
| Frank | 69320 | | 42 | | 2.12 | 1.19 | 0 |  |
| Garrett | 50587 | | 42 | | 2.29 | 1.49 | 0 |  |
| Gregory | 180441 | | 42 | | 2.43 | 1.27 | 0 |  |
| Hunter | 28551 | | 35 | | 2.09 | 1.42 | 0 |  |
| Ian | 81012 | | 34 | | 1.85 | 1.02 | 1 |  |
| Jack | 38637 | | 42 | | 2.05 | 1.29 | 0 |  |
| Jake | 31494 | | 41 | | 2.15 | 1.28 | 0 |  |
| Justin | 497467 | | 35 | | 2.14 | 1.17 | 0 |  |
| Kyle | 281151 | | 34 | | 2.32 | 1.43 | 0 |  |
| Logan | 43947 | | 42 | | 2.05 | 1.17 | 0 |  |
|  | Frequency | | *N* | | *M* | *SD* | *N* (neither) |  |
| Lucas | 55145 | | 42 | | 2.43 | 1.33 | 0 |  |
| Luke | 57349 | | 42 | | 2.38 | 1.29 | 0 |  |
| Matthew | 845816 | | 35 | | 2.34 | 1.21 | 0 |  |
| Nicholas | 441868 | | 35 | | 2.34 | 1.16 | 0 |  |
| Paul | 204262 | | 34 | | 1.94 | 0.98 | 0 |  |
| Peter | 113224 | | 34 | | 1.76 | 0.99 | 0 |  |
| Roger | 36950 | | 42 | | 2.05 | 1.15 | 0 |  |
| Ryan | 535094 | | 35 | | 2.11 | 1.16 | 0 |  |
| Scott | 219656 | | 42 | | 2.29 | 1.22 | 0 |  |
| Todd | 69238 | | 42 | | 2.17 | 1.25 | 0 |  |
| Zachary | 256606 | | 35 | | 2.14 | 1.14 | 0 |  |
| Infrequent American Male First Names | | | | | | | |  |
| Cale | 2612 | | 30 | | 3.17 | 1.60 | 5 |  |
| Dalton | 20532 | | 34 | | 3.44 | 1.94 | 1 |  |
| Donovan | 12581 | | 34 | | 3.88 | 2.11 | 1 |  |
| Duane | 11849 | | 35 | | 4.86 | 1.97 | 0 |  |
| Ernie | 2179 | | 35 | | 3.2 | 1.43 | 0 |  |
| Fletcher | 1458 | | 29 | | 3.24 | 1.94 | 4 |  |
| Frederic | 27290 | | 34 | | 3.03 | 1.59 | 1 |  |
| Jarrod | 16834 | | 35 | | 4.23 | 2.00 | 0 |  |
| Jimmie | 7655 | | 35 | | 2.94 | 1.41 | 0 |  |
| Marco | 23500 | | 31 | | 3.74 | 1.69 | 4 |  |
| Milo | 1131 | | 30 | | 2.90 | 1.49 | 5 |  |
| Norris | 1525 | | 29 | | 3.45 | 1.74 | 5 |  |
| Randolph | 5836 | | 34 | | 3.18 | 1.59 | 1 |  |
| Reece | 2190 | | 33 | | 3.79 | 1.58 | 2 |  |
| Rickey | 9463 | | 35 | | 2.86 | 1.31 | 0 |  |
|  | Frequency | | *N* | | *M* | *SD* | *N* (neither) |  |
| Roel | 1397 | | 23 | | 4.91 | 1.68 | 11 |  |
| Ronnie | 2144 | | 34 | | 3.18 | 1.53 | 1 |  |
| Rueben | 1172 | | 32 | | 4.22 | 1.79 | 3 |  |
| Wayne | 29907 | | 34 | | 3.24 | 1.60 | 0 |  |
| Wilfred | 1630 | | 33 | | 3.79 | 1.93 | 2 |  |

*Note.* Names were rated for stereotypicality on a 7-point scale (1 = “Very common for Whites”, 4 = “Both”, 7 = “Very common for Blacks” with the additional independent response option “neither”). *Frequency* refers to absolute frequency of male names based on Social Security Data from 1975-1995. *N* refers to the number of participants, who rated each name. N (neither) refers to the number of participants, who rated the name as “neither Black nor White”.

Table S7

*Name-to-face assignment within the stimulus sets for Pilot study and Studies 1, 4 and 6.*

| Set 1 | | Set 2 | | Set 3 | | Set 4 | | |
| --- | --- | --- | --- | --- | --- | --- | --- | --- |
| White names and Black faces | | | | | | | |  |
| Brad | BM 001 | Dylan | BM 030 | Matthew | BM 001 | Ian | BM 002 | |
| Hunter | BM 002 | Douglas | BM 210 | Andrew | BM 005 | Brett | BM 009 | |
| Drew | BM 005 | Peter | BM 032 | Ryan | BM 010 | Frank | BM 011 | |
| Connor | BM 009 | Edward | BM 033 | Justin | BM 013 | Todd | BM 015 | |
| Jake | BM 010 | Bradley | BM 034 | Brian | BM 016 | Dennis | BM 017 | |
| Arthur | BM 011 | Dustin | BM 036 | Nicholas | BM 018 | Luke | BM 019 | |
| Roger | BM 013 | Cody | BM 037 | Brandon | BM 020 | Lucas | BM 021 | |
| Colin | BM 015 | Gregory | BM 038 | Adam | BM 022 | Alan | BM 023 | |
| Jack | BM 016 | Paul | BM 039 | Kyle | BM 028 | Garrett | BM 025 | |
| Logan | BM 017 | Scott | BM 040 | Zachary | BM 026 | Ethan | BM 029 | |
| Black names and Black faces | | | | | | | |  |
| Malik | BM 018 | Lavon | BM 041 | Tyrone | BM 030 | Deshawn | BM 210 | |
| Marcellus | BM 019 | Kareem | BM 043 | Jermaine | BM 032 | Tyree | BM 033 | |
| Demetrius | BM 020 | Donte | BM 045 | Jamal | BM 034 | Trevon | BM 036 | |
| Deion | BM 021 | Lamont | BM 046 | Deandre | BM 037 | Rasheed | BM 038 | |
| Tremayne | BM 022 | Lamar | BM 200 | Marquis | BM 039 | Hakeem | BM 040 | |
| Hakeem | BM 023 | Marquis | BM 201 | Lamar | BM 041 | Tremayne | BM 043 | |
| Rasheed | BM 028 | Deandre | BM 202 | Lamont | BM 045 | Deion | BM 046 | |
| Trevon | BM 025 | Jamal | BM 203 | Donte | BM 200 | Demetrius | BM 201 | |
| Tyree | BM 026 | Jermaine | BM 205 | Kareem | BM 202 | Marcellus | BM 203 | |
| Deshawn | BM 029 | Tyrone | BM 207 | Lavon | BM 205 | Malik | BM 207 | |
| White names and White faces | | | | | | | |  |
| Ethan | WM 001 | Zachary | WM 028 | Scott | WM 001 | Logan | WM 002 | |
| Garrett | WM 002 | Kyle | WM 029 | Paul | WM 003 | Jack | WM 004 | |
| Alan | WM 003 | Adam | WM 031 | Gregory | WM 006 | Colin | WM 009 | |
| Lucas | WM 004 | Brandon | WM 032 | Cody | WM 013 | Roger | WM 011 | |
| Luke | WM 006 | Nicholas | WM 033 | Dustin | WM 012 | Arthur | WM 015 | |
| Dennis | WM 009 | Brian | WM 034 | Bradley | WM 016 | Jake | WM 017 | |
| Todd | WM 013 | Justin | WM 035 | Edward | WM 018 | Connor | WM 019 | |
| Frank | WM 011 | Ryan | WM 036 | Peter | WM 020 | Drew | WM 021 | |
| Brett | WM 012 | Andrew | WM 040 | Douglas | WM 023 | Hunter | WM 024 | |
| Ian | WM 015 | Matthew | WM 200 | Dylan | WM 214 | Brad | WM 218 | |
| Infrequent names and White faces | | | | | | | |  |
| Rickey | WM 016 | Dalton | WM 202 | Roel | WM 028 | Wayne | WM 029 | |
| Milo | WM 017 | Norris | WM 203 | Duane | WM 031 | Fletcher | WM 032 | |
| Jimmie | WM 018 | Marco | WM 204 | Jarrod | WM 033 | Ernie | WM 034 | |
| Frederic | WM 019 | Wilfried | WM 205 | Rueben | WM 035 | Randolph | WM 036 | |
| Cale | WM 020 | Reece | WM 206 | Donovan | WM 040 | Ronnie | WM 200 | |
| Ronnie | WM 021 | Donovan | WM 208 | Reece | WM 202 | Cale | WM 203 | |
| Randolph | WM 023 | Rueben | WM 209 | Wilfried | WM 204 | Frederic | WM 205 | |
| Ernie | WM 024 | Jarrod | WM 210 | Marco | WM 206 | Jimmie | WM 208 | |
| Fletcher | WM 214 | Duane | WM 212 | Norris | WM 209 | Milo | WM 210 | |
| Wayne | WM 218 | Roel | WM 213 | Dalton | WM 212 | Rickey | WM 213 | |

*Note.* Each of the four sets consist of 10 Black faces combined with typical Black American male names, 10 Black faces combined with typical White American male names, 10 White faces combined with typical White American male names, and 10 White faces combined with infrequent American male names. Faces codes refer to the original faces from the Chicago Face Database (Ma, Correll, & Wittenbrink, 2015).

Table S8

*List of female first names used in Studies 2 and 5 with frequencies based on Social Security Data from 1975-1995 and descriptive statistics of stereotypicality ratings.*

|  |  | Stereotypicality | | | |
| --- | --- | --- | --- | --- | --- |
|  | Frequency | *N* | *M* | *SD* | *N* (neither) |
| Typical Black American Female First Names | | | | | |
| Aiesha | 886 | 31 | 6.52 | 0.77 | 1 |
| Ebony | 31196 | 29 | 6.76 | 0.69 | 0 |
| Keisha | 14686 | 29 | 6.41 | 1.18 | 1 |
| Lakisha | 10541 | 31 | 6.61 | 1.02 | 0 |
| Lashandra | 787 | 29 | 6.62 | 0.86 | 1 |
| Lashelle | 546 | 31 | 6.61 | 0.92 | 0 |
| Lashonda | 6830 | 30 | 6.70 | 0.53 | 0 |
| Latisha | 14576 | 29 | 6.76 | 0.44 | 0 |
| Latoya | 42293 | 29 | 6.52 | 0.99 | 1 |
| Malika | 2167 | 30 | 6.43 | 0.86 | 1 |
| Shaniqua | 4578 | 30 | 6.67 | 0.66 | 0 |
| Shanise | 1039 | 28 | 6.46 | 0.79 | 1 |
| Shante | 4747 | 28 | 6.54 | 0.74 | 2 |
| Shavonne | 2337 | 29 | 6.41 | 0.91 | 0 |
| Tameisha | 315 | 28 | 6.54 | 1.10 | 1 |
| Tamika | 18236 | 29 | 6.55 | 0.87 | 0 |
| Tanisha | 13795 | 30 | 6.53 | 0.86 | 0 |
| Tashika | 520 | 30 | 6.47 | 0.90 | 1 |
| Tawanda | 2233 | 27 | 6.59 | 1.15 | 2 |
| Temeka | 1213 | 29 | 6.52 | 0.83 | 2 |
| Allison | 126995 | 31 | 1.71 | 1.01 | 0 |
| Amanda | 612846 | 30 | 1.90 | 1.09 | 0 |
|  | Frequency | *N* | *M* | *SD* | *N* (neither) |
| Typical White American Female First Names | | | | | |
| Amber | 272849 | 30 | 2.10 | 1.03 | 0 |
| Amy | 322505 | 29 | 1.93 | 1.10 | 0 |
| Anne | 43751 | 29 | 2.03 | 1.40 | 0 |
| Betsy | 8013 | 29 | 1.93 | 1.31 | 0 |
| Caitlin | 67738 | 30 | 2.03 | 1.03 | 1 |
| Carly | 28611 | 29 | 1.93 | 1.41 | 1 |
| Claire | 25250 | 29 | 1.79 | 1.05 | 0 |
| Colleen | 39118 | 29 | 1.83 | 1.23 | 1 |
| Ellen | 23256 | 31 | 1.58 | 0.96 | 0 |
| Emily | 298722 | 31 | 1.71 | 0.94 | 0 |
| Emma | 34765 | 30 | 1.70 | 0.88 | 0 |
| Hannah | 112545 | 30 | 1.90 | 1.12 | 0 |
| Heather | 364026 | 30 | 1.73 | 1.17 | 0 |
| Holly | 94703 | 31 | 2.10 | 1.22 | 0 |
| Jenna | 66417 | 29 | 1.90 | 1.26 | 0 |
| Jill | 56145 | 27 | 1.59 | 0.75 | 1 |
| Julia | 73677 | 30 | 1.77 | 1.01 | 0 |
| Katherine | 192512 | 31 | 1.71 | 1.07 | 0 |
| Kathy | 16430 | 29 | 2.00 | 1.13 | 0 |
| Katie | 120088 | 30 | 1.70 | 0.88 | 0 |
| Kristin | 110968 | 29 | 1.86 | 0.99 | 0 |
| Lauren | 261949 | 29 | 1.93 | 1.19 | 0 |
| Madeline | 23018 | 29 | 1.86 | 1.16 | 0 |
| Madison | 31256 | 29 | 1.93 | 1.13 | 1 |
| Mary | 197541 | 31 | 2.00 | 1.32 | 0 |
| Megan | 287530 | 29 | 1.83 | 0.93 | 0 |
|  | Frequency | *N* | *M* | *SD* | *N* (neither) |
| Meredith | 35936 | 31 | 1.65 | 0.98 | 0 |
| Molly | 58465 | 30 | 1.67 | 0.88 | 0 |
| Paige | 42747 | 31 | 1.58 | 0.76 | 0 |
| Peggy | 7340 | 30 | 1.57 | 0.97 | 0 |
| Rebecca | 280735 | 30 | 1.83 | 1.09 | 0 |
| Samantha | 282978 | 30 | 2.00 | 1.08 | 0 |
| Sarah | 500339 | 29 | 1.79 | 0.98 | 0 |
| Stephanie | 409124 | 31 | 2.10 | 1.16 | 0 |
| Susan | 68647 | 30 | 1.93 | 1.20 | 0 |
| Taylor | 108984 | 29 | 2.07 | 1.03 | 0 |
| Victoria | 136606 | 30 | 2.07 | 1.20 | 0 |
| Wendy | 58827 | 31 | 1.94 | 1.09 | 0 |
| Infrequent American Female First Names | | | | | |
| Alana | 13700 | 30 | 3.23 | 1.59 | 0 |
| Allie | 4581 | 30 | 2.17 | 1.56 | 0 |
| Brianne | 14698 | 29 | 2.52 | 1.77 | 1 |
| Cambria | 887 | 21 | 4.95 | 1.86 | 9 |
| Cornelia | 1038 | 23 | 4.39 | 2.27 | 7 |
| Elyse | 6818 | 28 | 3.00 | 1.81 | 3 |
| Gilian | 41809 | 28 | 2.64 | 1.99 | 3 |
| Gitty | 520 | 14 | 5.00 | 1.71 | 21 |
| Grace | 31240 | 29 | 2.62 | 1.57 | 1 |
| Helen | 18214 | 30 | 1.90 | 1.27 | 0 |
| Ivonne | 2330 | 27 | 5.30 | 1.73 | 2 |
| Kaylee | 14570 | 30 | 2.60 | 1.52 | 0 |
| Keva | 546 | 24 | 5.50 | 1.69 | 8 |
| Lara | 10532 | 29 | 2.41 | 1.40 | 0 |
|  | Frequency | *N* | *M* | *SD* | *N* (neither) |
| Lauran | 786 | 26 | 2.88 | 1.80 | 4 |
| Lillie | 2248 | 30 | 2.50 | 1.74 | 1 |
| Lizeth | 2167 | 20 | 4.65 | 2.18 | 11 |
| Marcey | 315 | 27 | 3.33 | 1.54 | 3 |
| Tammie | 4738 | 29 | 2.86 | 1.73 | 2 |
| Tanna | 1213 | 25 | 5.60 | 1.44 | 8 |

*Note.* Names were rated for stereotypicality on a 7-point scale (1 = “Very common for Whites”, 4 = “Both”, 7 = “Very common for Blacks”, with the additional independent response option “neither”). *Frequency* refers to absolute frequency of male names based on Social Security Data from 1975-1995. *N* refers to the number of participants, who rated each name. N (neither) refers to the number of participants, who rated the name as “neither Black nor White”.

Table S9

*Name-to-face assignment within the stimulus sets for Studies 2 and 5.*

| Set 1 | | Set 2 | | Set 3 | | Set 4 | |
| --- | --- | --- | --- | --- | --- | --- | --- |
| White names and Black faces | | | | | | | |
| Peggy | BF-001 | Betsy | BF-021 | Amanda | BF-001 | Sarah | BF-002 |
| Kathy | BF-002 | Madeline | BF-023 | Stephanie | BF-003 | Amy | BF-004 |
| Ellen | BF-003 | Claire | BF-024 | Heather | BF-005 | Megan | BF-006 |
| Carly | BF-004 | Emma | BF-025 | Emily | BF-007 | Rebecca | BF-008 |
| Madison | BF-005 | Colleen | BF-027 | Samantha | BF-009 | Amber | BF-010 |
| Meredith | BF-006 | Anne | BF-028 | Lauren | BF-011 | Mary | BF-012 |
| Paige | BF-007 | Jill | BF-029 | Katherine | BF-013 | Victoria | BF-014 |
| Molly | BF-008 | Wendy | BF-030 | Allison | BF-015 | Katie | BF-016 |
| Jenna | BF-009 | Caitlin | BF-031 | Hannah | BF-017 | Kristin | BF-018 |
| Susan | BF-010 | Julia | BF-032 | Taylor | BF-019 | Holly | BF-020 |
| Black names and Black faces | | | | | | | |
| Tameisha | BF-011 | Tashika | BF-033 | Latoya | BF-021 | Ebony | BF-023 |
| Lashelle | BF-012 | Lashandra | BF-034 | Tamika | BF-024 | Keisha | BF-025 |
| Aiesha | BF-013 | Shanise | BF-035 | Latisha | BF-027 | Tanisha | BF-028 |
| Temeka | BF-014 | Malika | BF-036 | Lakisha | BF-029 | Lashonda | BF-030 |
| Tawanda | BF-015 | Shavonne | BF-037 | Shante | BF-031 | Shaniqua | BF-032 |
| Shaniqua | BF-016 | Shante | BF-031 | Shavonne | BF-033 | Tawanda | BF-034 |
| Lashonda | BF-017 | Lakisha | BF-040 | Malika | BF-035 | Temeka | BF-036 |
| Tanisha | BF-018 | Latisha | BF-041 | Shanise | BF-037 | Aiesha | BF-039 |
| Keisha | BF-019 | Tamika | BF-042 | Lashandra | BF-040 | Lashelle | BF-041 |
| Ebony | BF-020 | Latoya | BF-043 | Tashika | BF-042 | Tameisha | BF-043 |
| White names and White faces | | | | | | | |
| Holly | WF-001 | Taylor | WF-023 | Julia | WF-001 | Susan | WF-002 |
| Kristin | WF-002 | Hannah | WF-024 | Caitlin | WF-003 | Jenna | WF-005 |
| Katie | WF-003 | Allison | WF-025 | Wendy | WF-006 | Molly | WF-007 |
| Victoria | WF-005 | Katherine | WF-026 | Jill | WF-008 | Paige | WF-009 |
| Mary | WF-006 | Lauren | WF-027 | Anne | WF-010 | Meredith | WF-011 |
| Amber | WF-007 | Samantha | WF-028 | Colleen | WF-012 | Madison | WF-013 |
| Rebecca | WF-008 | Emily | WF-029 | Emma | WF-014 | Carly | WF-015 |
| Megan | WF-009 | Heather | WF-030 | Claire | WF-017 | Ellen | WF-018 |
| Amy | WF-010 | Stephanie | WF-031 | Madeline | WF-019 | Kathy | WF-020 |
| Sarah | WF-011 | Amanda | WF-033 | Betsy | WF-021 | Peggy | WF-022 |
| Infrequent names and White faces | | | | | | | |
| Marcey | WF-012 | Gitty | WF-034 | Gilian | WF-023 | Grace | WF-024 |
| Keva | WF-013 | Lauran | WF-035 | Helen | WF-025 | Brianne | WF-026 |
| Cambria | WF-014 | Cornelia | WF-036 | Kaylee | WF-027 | Alana | WF-028 |
| Tanna | WF-015 | Lizeth | WF-037 | Lara | WF-029 | Elyse | WF-030 |
| Lilie | WF-017 | Ivonne | WF-038 | Tammie | WF-037 | Allie | WF-033 |
| Allie | WF-018 | Tammie | WF-039 | Ivonne | WF-034 | Lilie | WF-035 |
| Elyse | WF-019 | Lara | WF-200 | Lizeth | WF-036 | Tanna | WF-037 |
| Alana | WF-020 | Kaylee | WF-201 | Cornelia | WF-038 | Cambria | WF-039 |
| Brianne | WF-021 | Helen | WF-202 | Lauran | WF-200 | Keva | WF-201 |
| Grace | WF-022 | Gilian | WF-203 | Gitty | WF-202 | Marcey | WF-203 |

*Note.* Each of the four sets consist of 10 Black faces combined with typical Black American female names, 10 Black faces combined with typical White American female names, 10 White faces combined with typical White American female names, and 10 White faces combined with infrequent American female names.

Table S10

*List of male first names used in Study 3 with frequencies based on Social Security Data from 1975-1995 and descriptive statistics of stereotypicality ratings.*

|  |  | Stereotypicality | | | |
| --- | --- | --- | --- | --- | --- |
|  | Frequency | *N* | *M* | *SD* | *N* (neither) |
| Typical Chinese Male First Names | | | | | |
| Chang | 206 | 31 | 6.90 | 0.31 | 1 |
| Chao | 233 | 29 | 6.82 | 0.39 | 2 |
| Cheng | 448 | 30 | 6.83 | 0.47 | 2 |
| Chung | 178 | 30 | 6.90 | 0.31 | 2 |
| Dong | 210 | 30 | 6.45 | 1.55 | 3 |
| Fong | 259 | 31 | 6.83 | 0.46 | 1 |
| Hao | 170 | 31 | 6.83 | 0.46 | 2 |
| Huan | 63 | 31 | 6.67 | 0.71 | 1 |
| Jian | 60 | 31 | 6.60 | 1.04 | 1 |
| Jun | 433 | 31 | 6.60 | 0.67 | 1 |
| Kong | 701 | 29 | 6.71 | 0.53 | 3 |
| Luan | 151 | 30 | 6.31 | 1.31 | 2 |
| Meng | 437 | 28 | 6.70 | 0.61 | 3 |
| Peng | 209 | 29 | 6.71 | 0.60 | 3 |
| Wang | 65 | 31 | 6.83 | 0.46 | 1 |
| Won | 78 | 29 | 6.68 | 0.61 | 2 |
| Xiong | 163 | 30 | 6.86 | 0.44 | 2 |
| Yan | 180 | 30 | 6.83 | 0.47 | 2 |
| Yang | 158 | 31 | 6.97 | 0.18 | 2 |
| Yong | 167 | 29 | 6.86 | 0.45 | 3 |
| Adam | 327580 | 31 | 1.43 | 0.82 | 1 |
| Alan | 52888 | 31 | 1.43 | 0.90 | 1 |
|  | Frequency | *N* | *M* | *SD* | *N* (neither) |
| Typical White American Male First Names | | | | | |
| Andrew | 537371 | 30 | 1.34 | 0.86 | 1 |
| Brad | 28519 | 31 | 1.23 | 0.50 | 1 |
| Bradley | 132494 | 30 | 1.28 | 0.53 | 2 |
| Brandon | 438807 | 30 | 1.45 | 0.99 | 1 |
| Brett | 77176 | 30 | 1.31 | 0.76 | 2 |
| Brian | 466816 | 31 | 1.40 | 0.89 | 1 |
| Cody | 173611 | 31 | 1.27 | 0.58 | 1 |
| Colin | 38121 | 31 | 1.17 | 0.53 | 1 |
| Connor | 31302 | 31 | 1.20 | 0.48 | 1 |
| Dennis | 63849 | 31 | 1.33 | 0.66 | 1 |
| Douglas | 83845 | 31 | 1.17 | 0.38 | 1 |
| Drew | 29457 | 30 | 1.31 | 0.60 | 2 |
| Dustin | 157079 | 30 | 1.24 | 0.51 | 2 |
| Dylan | 83202 | 31 | 1.17 | 0.46 | 1 |
| Edward | 125444 | 31 | 1.37 | 0.76 | 1 |
| Ethan | 44897 | 31 | 1.27 | 0.58 | 1 |
| Frank | 69320 | 29 | 1.29 | 0.71 | 2 |
| Garrett | 50587 | 29 | 1.29 | 0.71 | 3 |
| Hunter | 28551 | 31 | 1.37 | 0.81 | 1 |
| Jack | 38637 | 31 | 1.27 | 0.52 | 1 |
| Jacob | 312669 | 31 | 1.20 | 0.55 | 1 |
| Jake | 31494 | 30 | 1.21 | 0.49 | 1 |
| Justin | 497467 | 31 | 1.50 | 1.22 | 1 |
| Kyle | 281151 | 31 | 1.37 | 0.89 | 1 |
| Logan | 43947 | 30 | 1.31 | 0.66 | 2 |
| Luke | 57349 | 31 | 1.30 | 0.75 | 1 |
|  | Frequency | *N* | *M* | *SD* | *N* (neither) |
| Matthew | 845816 | 30 | 1.34 | 0.77 | 1 |
| Mitchell | 57517 | 30 | 1.21 | 0.49 | 2 |
| Paul | 204262 | 31 | 1.40 | 0.89 | 1 |
| Peter | 113224 | 31 | 1.43 | 0.94 | 1 |
| Robert | 633713 | 31 | 1.33 | 0.76 | 1 |
| Roger | 36950 | 31 | 1.27 | 0.58 | 1 |
| Ryan | 535094 | 31 | 1.20 | 0.66 | 1 |
| Scott | 219656 | 30 | 1.21 | 0.49 | 2 |
| Spencer | 40941 | 30 | 1.41 | 0.78 | 2 |
| Stephen | 216626 | 31 | 1.47 | 1.01 | 1 |
| Todd | 69238 | 31 | 1.43 | 0.90 | 1 |
| Victor | 70619 | 29 | 1.57 | 0.96 | 3 |
| Infrequent American Male First Names | | | | | |
| Albin | 178 | - | - | - | - |
| Arne | 202 | - | - | - | - |
| Brach | 36 | - | - | - | - |
| Hyrum | 447 | - | - | - | - |
| Jese | 163 | - | - | - | - |
| Keil | 230 | - | - | - | - |
| Kjell | 206 | - | - | - | - |
| Klaus | 212 | - | - | - | - |
| Kolt | 259 | - | - | - | - |
| Larsen | 65 | - | - | - | - |
| Lydon | 60 | - | - | - | - |
| Marius | 456 | - | - | - | - |
| Mart | 78 | - | - | - | - |
| Olaf | 207 | - | - | - | - |
|  | Frequency | *N* | *M* | *SD* | *N* (neither) |
| Olan | 170 | - | - | - | - |
| Olen | 434 | - | - | - | - |
| Rees | 151 | - | - | - | - |
| Rhys | 699 | - | - | - | - |
| Sander | 167 | - | - | - | - |
| Tarl | 158 | - | - | - | - |

*Note.* Names were rated for stereotypicality on a 7-point scale (1 = “Very common for Whites”, 4 = “Both”, 7 = “Very common for Blacks”, and the additional independent response option “neither”). *Frequency* refers to absolute frequency of male names based on Social Security Data from 1975-1995. *N* refers to the number of participants, who rated each name. N (neither) refers to the number of participants, who rated the name as “neither Black nor White”.

Table S11

*Name-to-face assignment within the stimulus sets for Study 3.*

| Set 1 | | | Set 2 | | Set 3 | | Set 4 | | | |
| --- | --- | --- | --- | --- | --- | --- | --- | --- | --- | --- |
| White names and Chinese faces | | | | | | | | | |  |
| Colin | CFD-AM-225-102-N | Yan | | CFD-AM-201-076-N | Jian | CFD-AM-225-102-N | Yan | CFD-AM-225-102-N | | |
| Douglas | CFD-AM-227-184-N | Chang | | CFD-AM-203-086-N | Huan | CFD-AM-227-184-N | Chang | CFD-AM-227-184-N | | |
| Dylan | CFD-AM-228-214-N | Peng | | CFD-AM-206-086-N | Wang | CFD-AM-228-214-N | Peng | CFD-AM-228-214-N | | |
| Connor | CFD-AM-229-224-N | Dong | | CFD-AM-207-108-N | Won | CFD-AM-229-224-N | Dong | CFD-AM-229-224-N | | |
| Jacob | CFD-AM-230-150-N | Chao | | CFD-AM-209-048-N | Luan | CFD-AM-230-150-N | Chao | CFD-AM-230-150-N | | |
| Ryan | m-011-01 | Fong | | CFD-AM-210-035-N | Yang | m-011-01 | Fong | m-011-01 | | |
| Jake | m-013-01 | Jun | | CFD-AM-213-056-N | Xiong | m-013-01 | Jun | m-013-01 | | |
| Mitchell | m-014-01 | Meng | | CFD-AM-218-085-N | Yong | m-014-01 | Meng | m-014-01 | | |
| Scott | m-015-01 | Cheng | | CFD-AM-219-101-N | Hao | m-015-01 | Cheng | m-015-01 | | |
| Brad | m-022-01 | Kong | | CFD-AM-223-138-N | Chung | m-022-01 | Kong | m-022-01 | | |
| Chinese names and Chinese faces | | | | | | | | |  |  |
| Jian | CFD-AM-201-076-N | Yan | | CFD-AM-225-102-N | Colin | CFD-AM-201-076-N | Dustin | CFD-AM-201-076-N | | |
| Huan | CFD-AM-203-086-N | Chang | | CFD-AM-227-184-N | Douglas | CFD-AM-203-086-N | Cody | CFD-AM-203-086-N | | |
| Wang | CFD-AM-206-086-N | Peng | | CFD-AM-228-214-N | Dylan | CFD-AM-206-086-N | Ethan | CFD-AM-206-086-N | | |
| Won | CFD-AM-207-108-N | Dong | | CFD-AM-229-224-N | Connor | CFD-AM-207-108-N | Jack | CFD-AM-207-108-N | | |
| Luan | CFD-AM-209-048-N | Chao | | CFD-AM-230-150-N | Jacob | CFD-AM-209-048-N | Roger | CFD-AM-209-048-N | | |
| Yang | CFD-AM-210-035-N | Fong | | m-011-01 | Ryan | CFD-AM-210-035-N | Bradley | CFD-AM-210-035-N | | |
| Xiong | CFD-AM-213-056-N | Jun | | m-013-01 | Jake | CFD-AM-213-056-N | Frank | CFD-AM-213-056-N | | |
| Yong | CFD-AM-218-085-N | Meng | | m-014-01 | Mitchell | CFD-AM-218-085-N | Garrett | CFD-AM-218-085-N | | |
| Hao | CFD-AM-219-101-N | Cheng | | m-015-01 | Scott | CFD-AM-219-101-N | Luke | CFD-AM-219-101-N | | |
| Chung | CFD-AM-223-138-N | Kong | | m-022-01 | Brad | CFD-AM-223-138-N | Brett | CFD-AM-223-138-N | | |
| White names and White faces | | | | | | | | | |  |
| Drew | CFD-WM-001-014-N | Paul | | CFD-WM-001-014-N | Drew | CFD-WM-028-003-N | Paul | CFD-WM-028-003-N | | |
| Logan | CFD-WM-004-010-N | Spencer | | CFD-WM-004-010-N | Logan | CFD-WM-031-003-N | Spencer | CFD-WM-031-003-N | | |
| Dennis | CFD-WM-006-002-N | Adam | | CFD-WM-006-002-N | Dennis | CFD-WM-033-025-N | Adam | CFD-WM-033-025-N | | |
| Robert | CFD-WM-009-002-N | Alan | | CFD-WM-009-002-N | Robert | CFD-WM-034-030-N | Alan | CFD-WM-034-030-N | | |
| Andrew | CFD-WM-011-002-N | Peter | | CFD-WM-011-002-N | Andrew | CFD-WM-200-034-N | Peter | CFD-WM-200-034-N | | |
| Matthew | CFD-WM-015-002-N | Todd | | CFD-WM-015-002-N | Matthew | CFD-WM-203-023-N | Todd | CFD-WM-203-023-N | | |
| Edward | CFD-WM-016-001-N | Brandon | | CFD-WM-016-001-N | Edward | CFD-WM-204-031-N | Brandon | CFD-WM-204-031-N | | |
| Hunter | CFD-WM-020-001-N | Stephen | | CFD-WM-020-001-N | Hunter | CFD-WM-205-007-N | Stephen | CFD-WM-205-007-N | | |
| Kyle | CFD-WM-021-001-N | Justin | | CFD-WM-021-001-N | Kyle | CFD-WM-206-045-N | Justin | CFD-WM-206-045-N | | |
| Brian | CFD-WM-024-015-N | Victor | | CFD-WM-024-015-N | Brian | CFD-WM-208-068-N | Victor | CFD-WM-208-068-N | | |
| Infrequent names and White faces | | | | | | | | | |  |
| Lydon | CFD-WM-028-003-N | Kjell | | CFD-WM-028-003-N | Lydon | CFD-WM-001-014-N | Kjell | CFD-WM-001-014-N | | |
| Brach | CFD-WM-031-003-N | Arne | | CFD-WM-031-003-N | Brach | CFD-WM-004-010-N | Arne | CFD-WM-004-010-N | | |
| Larsen | CFD-WM-033-025-N | Olaf | | CFD-WM-033-025-N | Larsen | CFD-WM-006-002-N | Olaf | CFD-WM-006-002-N | | |
| Mart | CFD-WM-034-030-N | Klaus | | CFD-WM-034-030-N | Mart | CFD-WM-009-002-N | Klaus | CFD-WM-009-002-N | | |
| Rees | CFD-WM-200-034-N | Keil | | CFD-WM-200-034-N | Rees | CFD-WM-011-002-N | Keil | CFD-WM-011-002-N | | |
| Tarl | CFD-WM-203-023-N | Kolt | | CFD-WM-203-023-N | Tarl | CFD-WM-015-002-N | Kolt | CFD-WM-015-002-N | | |
| Jese | CFD-WM-204-031-N | Olen | | CFD-WM-204-031-N | Jese | CFD-WM-016-001-N | Olen | CFD-WM-016-001-N | | |
| Sander | CFD-WM-205-007-N | Hyrum | | CFD-WM-205-007-N | Sander | CFD-WM-020-001-N | Hyrum | CFD-WM-020-001-N | | |
| Olan | CFD-WM-206-045-N | Marius | | CFD-WM-206-045-N | Olan | CFD-WM-021-001-N | Marius | CFD-WM-021-001-N | | |
| Albin | CFD-WM-208-068-N | Rhys | | CFD-WM-208-068-N | Albin | CFD-WM-024-015-N | Rhys | CFD-WM-024-015-N | | |

*Note.* Each of the four sets consist of 10 Chinese faces combined with typical Chinese male names, 10 Chinese faces combined with typical White American male names, 10 White faces combined with typical White American male names, and 10 White faces combined with infrequent American male names.

**References**

Bates, D., Maechler, M., Bolker, B., & Walker, S. (2015). Fitting Linear Mixed-Effects Models Using lme4. *Journal of Statistical Software, 67*(1), 1-48. doi:10.18637/jss.v067.i01

Hancock, K. J., & Rhodes, G. (2008). Contact, configural coding and the other-race effect in face recognition. *British Journal of Psychology, 99*, 45-56. doi:10.1348/000712607x199981

Judd, C. M., Westfall, J., & Kenny, D. A. (2012). Treating stimuli as a random factor in social psychology: a new and comprehensive solution to a pervasive but largely ignored problem. *Journal of Personality and Social Psychology, 103*(1), 54. doi:10.1037/a0028347

Ma, D., Correll, J., & Wittenbrink, B. (2015). The Chicago Face Database: A Free Stimulus Set of Faces and Norming Data. *Behavior Research Methods, 47*, 1122-1135. doi:10.3758/s13428-014-0532-5

R Core Team. (2017). R: A language and environment for statistical computing. Vienna, Austria: R Foundation for Statistical Computing. Retrieved from <https://www.R-project.org/>
